# Mesenchymal Stem Cell Treatment Perspectives in Peripheral Nerve Regeneration: Systematic Review

**DOI:** 10.3390/ijms22020572

**Published:** 2021-01-08

**Authors:** Andrea Lavorato, Stefania Raimondo, Marina Boido, Luisa Muratori, Giorgia Durante, Fabio Cofano, Francesca Vincitorio, Salvatore Petrone, Paolo Titolo, Fulvio Tartara, Alessandro Vercelli, Diego Garbossa

**Affiliations:** 1 Neurosurgery Unit, Department of Neuroscience “Rita Levi Montalcini”, University of Turin, 10126 Turin, TO, Italy; fabio.cofano@gmail.com (F.C.); vincitorio.francesca@gmail.com (F.V.); svt.petrone@gmail.com (S.P.); dgarbossa@gmail.com (D.G.); 2 Department of Clinical and Biological Sciences, Neuroscience Institute Cavalieri Ottolenghi, University of Turin, 10043 Orbassano, TO, Italy; stefania.raimondo@unito.it (S.R.); luisa.muratori@unito.it (L.M.); 3 Department of Neuroscience “Rita Levi Montalcini”, Neuroscience Institute Cavalieri Ottolenghi, University of Turin, 10043 Orbassano, TO, Italy; marina.boido@unito.it (M.B.); alessandro.vercelli@unito.it (A.V.); 4 Faculty of Medicine and Surgery, University of Turin, 10126 Turin, TO, Italy; giorgiadurante.gd95@gmail.com; 5 Traumatology–Reconstructive Microsurgery, Department of Orthopaedics and Traumatology, CTO Hospital, 10126 Turin, TO, Italy; info@paolotitolo.it; 6 Neurosurgery Unit, Istituto Clinico Città Studi (ICCS), 20131 Milan, MI, Italy; tartarafulvio@gmail.com

**Keywords:** mesenchymal stem cells, nerve lesions, peripheral nerve regeneration, regenerative medicine

## Abstract

Traumatic peripheral nerve lesions affect hundreds of thousands of patients every year; their consequences are life-altering and often devastating and cause alterations in movement and sensitivity. Spontaneous peripheral nerve recovery is often inadequate. In this context, nowadays, cell therapy represents one of the most innovative approaches in the field of nerve repair therapies. The purpose of this systematic review is to discuss the features of different types of mesenchymal stem cells (MSCs) relevant for peripheral nerve regeneration after nerve injury. The published literature was reviewed following the Preferred Reporting Items for Systematic Reviews and Meta-Analyses (PRISMA) guidelines. A combination of the keywords “nerve regeneration”, “stem cells”, “peripheral nerve injury”, “rat”, and “human” were used. Additionally, a “MeSH” research was performed in PubMed using the terms “stem cells” and “nerve regeneration”. The characteristics of the most widely used MSCs, their paracrine potential, targeted stimulation, and differentiation potentials into Schwann-like and neuronal-like cells are described in this paper. Considering their ability to support and stimulate axonal growth, their remarkable paracrine activity, their presumed differentiation potential, their extremely low immunogenicity, and their high survival rate after transplantation, ADSCs appear to be the most suitable and promising MSCs for the recovery of peripheral nerve lesion. Clinical considerations are finally reported.

## 1. Introduction

Traumatic nerve lesions affect several hundred thousand patients every year in Europe and the USA [1]. Common causes of the most severe cases include motor vehicle, domestic, work, and sport accidents [2]. The consequences of peripheral nerve injuries (PNIs) are notoriously life-altering and often devastating, resulting in various degrees of sensorimotor impairment of the affected limb [3,4]. Nonetheless, nerve repair may occur to a certain extent. In the attempt to predict the outcome of peripheral nerve repair, many factors must be considered, including type, location, and extent of nerve injury; timing of surgery; type of repair; proper alignment of fascicles; surgical technique; and patient comorbidities [5].

Spontaneous peripheral nerve recovery is often inadequate and strictly depends on the injury type and extension [2]: for high-energy traumatic injuries, recovery is negatively affected by the severity and nerve discontinuity [6]. The functional outcome can be limited by inflammation, scar formation, and misdirection of regenerating sensory and motor axons [7]. For nerve injuries with a gap, nerve autograft treatment could present many disadvantages such as biological complexity, donor site morbidity, limited length of graft tissue availability, and the requirement of multiple surgeries [8]. For these reasons, the currently approved therapies are not fully satisfactory [3].

The morbidity and devastating physical and psychological effects on patients encourage researchers to provide alternative and potentially more efficacious treatment/intervention modalities.

In this context, cell therapy represents one of the most innovative therapeutic approaches in the field of nerve repair [7,9]. In particular, the use of mesenchymal stem cells (MSCs) is of great interest in regenerative medicine since they can support damaged tissues by targeting differentiating processes that influence several changes in cell morphology, metabolic activity, secretion of growth factors, and signal responsiveness [7].

**Figure 1:**
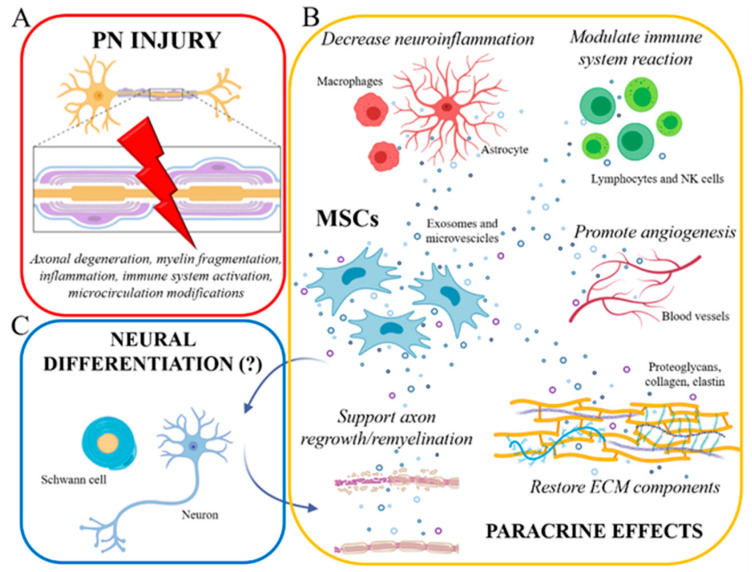
(**A**) Peripheral nerve injury triggers a cascade of cellular/molecular events, including axonal damage, myelin disruption, early inflammatory reactions, immune system activation, and microcirculation modifications. (**B**) Mesenchymal stem cells (MSCs), exerting their prominent paracrine role by exosome/microvesicle secretion, can effectively counteract these events: indeed, MSCs are able to modulate neuroinflammation and immune cell reaction, to promote new blood vessel formation (angiogenesis), to form ECM components, and to sustain axonal regrowth and remyelination. (**C**) Some authors also tried to develop in vitro differentiation protocols to induce neural (Schwann cells and neurons) differentiation of MSCs (eventually able to further support axonal regeneration/remyelination), Nevertheless, the real occurrence of such differentiation is still much debated. Created with BioRender software.

The cellular mechanisms by which MSCs exert their biological effects (Figure 1) are not completely understood, and their clinical applications in peripheral nerve repair are still in development. For this reason, MSCs have been widely considered for in vitro and in vivo studies to test their efficacy in supporting nerve regrowth [10].

Mesenchymal stem cells have a dual role: on one hand, under specific conditions, they can replace injured tissue cells; on the other hand, they can maximize the intrinsic regenerative capacity of injured tissue by producing growth factors and cytokines and by immunomodulating response after nerve injury [10].

Accordingly, Mathot et al. (2019) reported two main hypotheses regarding the mechanism of action of MSCs in tissue repair. The first is based on the secretion of trophic factors produced by MSCs that are important for remodeling of the extracellular matrix and tissue regeneration; these factors improve angiogenesis, inhibit scar tissue formation, and stimulate tissue regeneration. In addition findings from in vitro and in vivo studies also reported a key immunomodulatory role played by MSCs for tissue repair: after the first inflammatory response of the damaged tissue caused by injury, studies suggest that the pro-inflammatory cytokines produced by lymphocytes are able to activate MSCs which, on the other hand, decrease the aggressive immunological response carried out by NK cells through a feedback loop that result in decreased cytotoxicity [11,12,13].

In addition, Mathot and colleagues proposed that MSCs can transdifferentiate in vivo at the site of injury thanks to growth factors and paracrine molecules produced by the surrounding tissue that stimulates differentiation of MSCs into the required cell type (i.e., Schwann-like) [10].

All these mechanisms of action will be further described in Section 3.3.1, Section 3.3.2 and Section 3.3.3.

The purpose of this systematic review is to discuss the features of different types of MSCs relevant for peripheral nerve regeneration after nerve injuries. Particular attention has been paid to their mechanism of action, with a special mention to cell delivery modalities, and references preclinical and clinical data of each study.

## 2. Materials and Methods

### 2.1. Search Strategy

**Figure 2:**
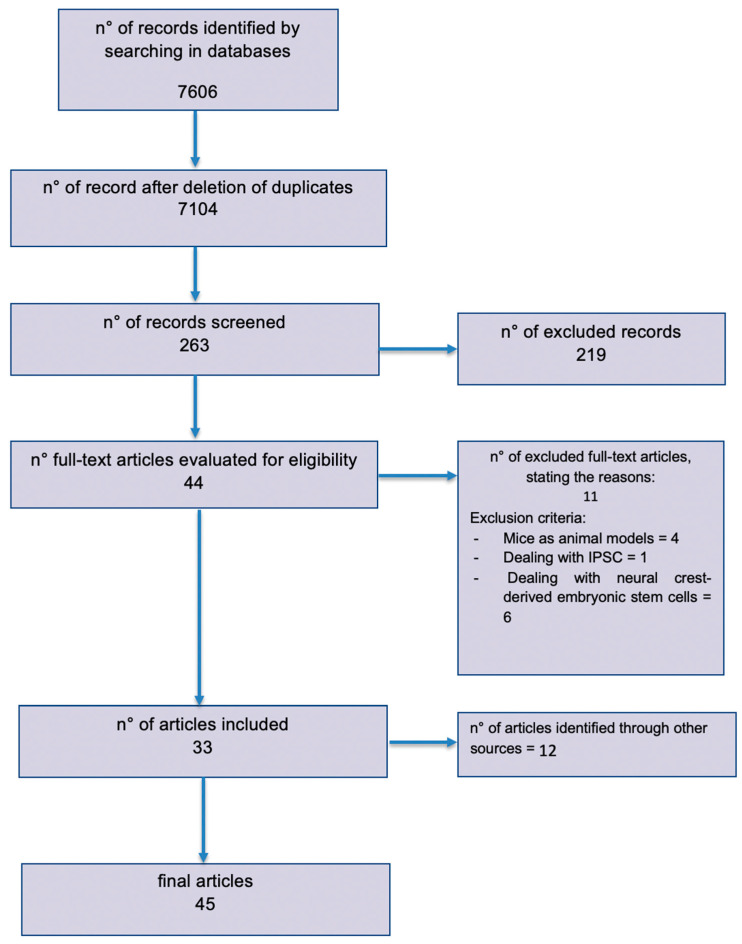
Preferred Reporting Items for Systematic Reviews and Meta-Analyses (PRISMA) flow diagram outlining the systematic review process.

The published literature was reviewed following the Preferred Reporting Items for Systematic Reviews and Meta-Analyses (PRISMA) guidelines [14] (Figure 2) (www.prisma-statement.org). A combination of the keywords “nerve regeneration”, “stem cells”, “peripheral nerve injury”, “rat”, and “human” was used. Additionally, a “MeSH” research was performed in PubMed using the terms “stem cells” and “nerve regeneration”.

The searches were completed in April 2020. Citation titles and abstracts were screened by the authors for prespecified selection criteria.

### 2.2. Study Selection and Eligibility Criteria

The authors included articles analyzing the current usage of MSCs in the treatment of peripheral nervous system (PNS) injury in humans and rats.

This screening was followed by a full-text assessment of the remaining articles. We finally reported all the articles dealing with PNS lesion injuries with MSC treatment.

A total of 44 items met our selection criteria. All articles were written in English. In addition, 8 articles were added in the drafting phase. They were included with the aim to deepen the amniotic stem cells, skeletal muscle stem cells, and nerve conduit topics.

Eleven articles were removed because they did not meet our inclusion criteria: four papers did not have rats as animal models, one paper took into account Induced Pluripotent Stem Cells (IPSCs), and six papers studied neural crest-derived embryonic stem cells or already differentiated neuronal cells (Figure 2).

## 3. Results

A total of 45 articles were finally included. Papers from 2006 through 2019 were considered for this review. The entire research process and studies’ selection are described in the PRISMA diagram (Figure 2).

We divided our articles according to the type of MSC considered (see Table 1). In addition, stem cell (SC) administration and growth factor release were analyzed in all studies and summarized in this review; finally, some clinical considerations are reported.

As indicated in Table 1, the most common are bone marrow-derived stem cells (BMSCs) (19 articles, including papers that use BMSCs alone or with MSCs from other sources) and adipose-derived stem cells (ADSCs) (18 articles, including papers that use ADSCs alone or with MSCs from other sources). BMSCS was historically the first source of MSCs studied. MCSs were collected from adipose tissue secondly in search for a more accessible and clinically feasible source. MSCs from other sources are described in this review: dental pulp stem cells (DPSCs) (7 articles), fetal stem cells (FetalSCs) amniotic (AMSCs)/umbilical cord (UCMSCs)—(11 articles), and skeletal muscle stem cells (SkSCs; 3 articles). For each source, our attention focuses on the processes of preparation, collection, and implantation. The effects of MSCs on nerve regeneration, methods of differentiation, and transplantation are also taken into account. The main characteristics of each MSC are reported in Table 2.

Four studies [19,20,24,31] dealt with differentiation modalities of MSCs into Schwann-like cells, and another one [34] analyzed the mechanism of MSC differentiation towards astrocytes.

The different outcomes and follow-up times were reported in the articles considered (Table 3). Regenerative capacity was evaluated after a period of time ranging from 2 [34] to 12 [15] weeks using different assessment techniques such as electromyography (EMG), functional tests, magnetic resonance imaging (MRI), immunohistochemistry, and immunohistological examination. Only one study [45] describes regenerative treatment with SCs following injury in humans in radial and median nerves. The trend of nerve regeneration was evaluated every 2 months for 3 years with MRI, EMG, and clinical assessment. The 3-year follow-up showed suitable functional recovery of the patient. It is important to highlight that the SCs used in this study are not MSCs since they are derived from the neural crests (skin-derived stem cells—SDSCs).

The remaining 12 articles were systematic reviews dealing with MSCs and nerve regeneration.

### 3.1. Mesenchymal Stem Cells

The use of MSCs in repairing PNS injuries is of great interest, as suggested by the recent literature, because of their fast self-proliferation, paracrine/autocrine activity, and presumed trans-differentiation potential [28] (Figure 1).

MSCs can be harvested from several sources: bone marrow (BMSCs), subcutaneous white adipose tissue (ADSCs), fetal tissues such as umbilical cord blood, Wharton jelly (UCMSCs), avascular amniotic mesoderm (AMSCs), dental pulp tissue of adult/permanent teeth (DPSCs), and skeletal muscles (SkSCs).

MSCs are able to secrete various paracrine factors with major functions, including reparative, antiapoptotic, anti-inflammatory, antioxidative, antifibrotic, and/or antibacterial effects [46]. These effects are due to responses to environmental cues and are considered essential for the therapeutic potential of the MSCs to repair damaged tissue.

Regarding the ability to promote peripheral nerve repair, MSCs are capable of secreting neurotrophins such as nerve growth factor (NGF), brain-derived neurotrophic factor (BDNF), glial-cell-line-derived neurotrophic factor (GDNF), and ciliary neurotrophic factor (CNTF) and trophic factors such as neuregulin-1 (NRG-1), that promote neuronal and glial response during the regenerative process.

Under particular conditions in vitro, all types of MSC could undergo targeted differentiation in neuronal or glial cell types; however, the ability of differentiated MSCs to improve nerve regeneration in vivo was evaluated only in a few studies focused on the application of UMSCs, AMSCs, and ADSCs that have been shown to sustain peripheral nerve regeneration after injury [16].

For this reason, the aim of this review is to analyze the main sources of MSCs and to study their possible application for repairing injuries of peripheral nerves.

#### 3.1.1. Bone Marrow-Derived Mesenchymal Stem Cell

Adult BMSC multipotent cells are able to differentiate into the mesoderm lineages (fat, bone, muscle, and cartilage). Some authors also report that, under particular conditions in vitro, they might be able to undergo neuronal or glial differentiation [7,38]. However, in vivo, in the case of PNS injury, these cells, undifferentiated, may in particular exert their functions through paracrine/autocrine activity [38].

Indeed, BMSCs effectively produce and secrete neurotrophins, such as nerve growth factor (NGF), brain-derived neurotrophic factor (BDNF), glial-cell-line-derived neurotrophic factor (GDNF), and ciliary neurotrophic factor (CNTF) [38].

Limitations in the use of BMSCs consist in the invasive extraction from bone marrow, and MSC sources such as adipose tissue display a simpler extraction procedure but a lower yield in comparison with other MSCs in addition to their decreased proliferative and differentiation capacity with patient’s age [7,32].

#### 3.1.2. Adipose Tissue-Derived Mesenchymal Stem Cell

Adipose-derived stem cells (ADSCs) are of particular interest since they can be easily harvested and show widespread availability. Indeed, the high level of subcutaneous adipose tissue in humans allows for isolation during conventional liposuction, overcoming tissue morbidity associated with bone marrow aspiration [24,28,30,32,37,38,39].

In addition, the amount of ADSCs in adipose tissues is 100- to 500-fold compared with that of BMSCs. In humans, two types of adipose tissue are identified: white vs. brown adipose tissue. Brown adipose tissue is abundant in newborns and plays a role in body thermoregulation, but it is minimal in adults, mainly located in the abdominal cavity surrounding the kidneys, making its harvest particularly difficult. For these reasons, subcutaneous white adipose tissue is the main source of ADSCs: moreover, it has a stronger antiapoptotic capacity than brown adipose tissue-derived SCs [33].

ADSCs display a higher yield and higher proliferative rates in culture compared with BMSCs, low immunogenicity [28], and the ability to improve the microenvironment for host neural regeneration by inhibiting inflammatory responses [40]. Indeed, in vitro studies have showed that these cells are able to produce specific mRNAs that promote the release of paracrine factors, including BDNF, glial-growth-like factor (GGF), neuregulin-1 (NRG-1), vascular endothelial growth factor (VEGF), hepatocyte growth factor (HGF), and insulin-like growth factor (IGF) that can promote neuronal regeneration modulating the lesion microenvironment [7,30,32,33,38].

In addition, it is widely accepted that ADSCs have great ability in multidirectional differentiation, including toward the Schwann-like cells [47]. The differentiation of ADSCs to a Schwann cell phenotype might have a beneficial role for the treatment of peripheral nerve injuries. Indeed, in vitro studies reported ADSC differentiation toward a Schwann cell phenotype, supporting the hypothesis that these cells can provide functional benefits for peripheral nerve repair [47].

#### 3.1.3. Fetal Tissue-Derived Mesenchymal Stem Cell

Umbilical cord and amniotic fluid (both considered fetal tissues) are the most primitive sources of MSCs [7].

Although these cells can be harvested with noninvasive procedures, during which genetic damage is not significant, and even if their proliferative profile is wide.

UCMSCs can be taken from both umbilical cord blood and Wharton jelly [38]. There are several potential advantages in using these particular FetalMSCs, including easy accessibility [40], the characteristic of which are immunologically inertness, the absence of ethical concerns with their use, and their low probability of resulting in graft-vs-host disease [16,38]. A few reports of tumorigenesis in transplantation experiments of UCMSCs and of UCMSC-derived cells have been published [7].

UCMSCs can synthesize trophic factors as glial cell line-derived neurotrophic factor (GDNF) [16], vascular endothelial growth factor (VEGF) [16], ciliary neurotrophic factor (CNF) [16], NGF [7], and BDNF [7].

Matsuse et al. [42] reported that UCMSCs did not express Schwann cell markers but, after induction by treatment with β-mercaptoethanol, retinoic acid, and specific cytokines, most UCMSCs became positive for several Schwann cell markers. Moreover, when transplanted in transected sciatic nerve rat models, they enhanced nerve regeneration.

Wharton’s jelly is an ideal, unique, easily accessible, and noncontroversial source for MSCs [7,41], having unique properties between embryonic and adult stem cells [41]. FetalSCs are able to secrete NGF, BDNF, and NT-3 and to stimulate neurites’ growth in vitro [7,40,41]. These particular cells show positive expression of surface markers for mesenchymal lineage, with the potential to differentiate into Schwann cell-like cells [41]. These cells are negative for histocompatibility complex (MHC) class II, have low expression of MHC class I (low immunogenicity) [40], and represent a noninvasive source of MSCs that could be used after birth of the donor, stored cryogenically [41].

AMSCs derive from the avascular amniotic mesoderm and are relatively non-immunogenic cells [38]. As for the other MSCs, Li et al. reported that the administration of AMSCs improves functional recovery after nerve injury because they can mediate neovascular trophism and exert neurotrophic effects: indeed, they secrete angiogenic factors (e.g., VEGF); can express chemokine genes and receptors (CCR2, CCR3, and CCR5); and can enhance cell migration, engraftment, and endothelial trans-differentiation properties [47]. While having the characteristics of mesenchymal stem cells, AMSCs can also apparently differentiate into neural tissue under specific in vitro conditions. Jiang et al. described the possibility to employ embryonic stem cells derived from the blastocyst stage of embryonic development. They are not generally used because of the difficulty of finding embryonic tissues due to the ethical dilemma of using a human embryo [7].

#### 3.1.4. Dental Pulp-Derived Mesenchymal Stem Cell

Human dental pulp represents a suitable stem cell source due to its easy accessibility through routine procedures of wisdom teeth extraction and houses a progenitor mesenchymal population able to differentiate into multi-lineage cells. Data from different studies suggest that these cells not only show self-renewal and multiple differentiation potential but also display immunomodulatory properties associated with the expression of interleukin-8 (IL-8), interleukin-6 (IL-6), and TGF-β [43]; they also have a promising regenerative capability towards tissue damage [48].

Moreover, when conveniently stimulated in vitro, they can express both neural and Schwann cell phenotype in vitro [7,38], including glial fibrillary acid protein (GFAP), nestin, βIII-tubulin, NF-200, and MAP-2 [42,43].

Different in vitro studies show that DPSCs can promote peripheral nerve recovery in association with conduits or electromagnetic fields after nerve injury [7,38,42,43].

#### 3.1.5. Skeletal Muscle-Derived Mesenchymal Stem Cell

Isolated skeletal muscle-derived stem cells (SkSCs) are able to differentiate into multiple lineages, including myogenic, adipogenic, and osteoblastic lineages [38]. They represent an opportunity in peripheral nerve regeneration—together with muscle atrophy prevention—to reconstruct the muscle–nerve–blood vessel unit [7,38].

### 3.2. Stem Cell Delivery

Stem cells can be delivered in the site of the nerve lesion through different techniques.

#### 3.2.1. Micro-Injection

MSC micro-injection has been described as a hypothetical method with potential good results. Local injection leads to a significant increase in axonal fiber counts, improves electromotor recovery, shows immune modulatory effects, synergistically supports local Schwann cells, and enhances nerve regeneration with functional recovery through the secretion of neuroprotective factors [8].

Despite these beneficial effects, this method presents different disadvantages that make the technique prejudicial. The high-pressure build-up in the syringe could lead to ultrastructural trauma, nonhomogeneous distribution of cells, and nonoptimal nerve regeneration. A micro-injection at the site of injury as well as reaching the epineurium could be less precise because of the size of the fibers, especially with the use of a large needle to avoid cell injury [7,10].

Intravenous injection of MSCs has been investigated as an alternative to MSC local injection to prevent possible nerve damage and cell leakage and focuses on the more likely trophic function of MSCs as a possible response to inflammatory chemokines and hypoxic conditions. However, despite the excellent trophism, these cells may not reach the site of lesion by capillary entrapment [2,10].

The study of Piñero et al. analyzed the effect of BMMCs (bone marrow mononuclear cells) transplanted in rats after sciatic nerve injury. The study does not employ MSCs, but it may be helpful to comprehend the outcomes of this procedure. BMMCs were harvested from GFP (Green Fluorescent Protein) adult rats and were at first characterized by analyzing the expression of the selected markers for multipotent progenitors and Schwann cells to validate their potential application in transplantation studies. The results of this study displayed that BMMCs, intravenously transplanted in adult wild type rats that have undergone a sciatic nerve crush injury, reached the injury site and interestingly express Schwann cells markers in injured sciatic nerve harvested 60 days after injury [21].

Lastly, Jiang et al. mentioned suspension of the stem cells in a fibrin matrix and its injection in the repair site as another method for the delivery of MSCs [7].

#### 3.2.2. Natural Nerve Conduits and Artificial Nerve Conduits

When primary closure is not possible, nerve conduits become an indispensable tool both to bridge the nerve gap and to deliver therapeutic cells to the site of injury. It then becomes important to examine nerve conduits not only as a standalone therapy but also as a potential platform for stem cell delivery [27].

Various materials have been used for the reconstruction of peripheral nerves injuries with substance loss. The most common ones are classified as natural or artificial. Natural conduits are derived from biological tissues such as veins, arteries, and muscle properly treated for clinical use. Artificial conduits are made of entirely laboratory-produced materials.

##### Natural Nerve Conduits

Various biological materials such as muscle or vessels can be used for nerve-gap repair thanks to their useful properties; they are rich in extracellular matrix (ECM) proteins such as collagen and laminin, and they provide a microenvironment that promotes cell adhesion, axonal guidance, and migration of nonneuronal cells [7,15,30]. In fact, even commercial natural conduits are usually filled with ECM components [7].

In addition to the adhesive and differentiating properties of ECM components, platelet-rich plasma or growth factors can be added to the natural conduits, thus encouraging differentiation in the reparative phenotype of Schwann cells and MSCs [3].

Natural vascular grafts, similar to artificial nerve conduits [49], have the potential for clinical feasibility in large nerve defects [32], especially vein autografts, which are more abundantly available and induce less donor-site morbidities compared with nerve autografts [50]. Nevertheless, they show no functional benefits compared with other nerve grafts [24].

We can also consider muscle tissue as a natural conduit: in fact, the three-dimensional environment provided by skeletal muscle basal lamina promotes cell adhesion to ECM [27,50]. For this reason, intramuscular injection of MSCs enhances nerve regeneration [10].

Intramuscular injection of MSCs demonstrated poorly fibrotic degeneration and good alignment in regenerating axons [27]. In addition, donor sites for muscle grafts are numerous. However, in longer nerve defects, the effectiveness of skeletal muscle autografts may be progressively reduced [50].

Stem cell injection into muscle-in-vein grafts used as nerve conduits has been successfully used to promote nerve regeneration [27], while the tendon autograft does not differ from a muscle graft in supporting axonal regeneration, so it can be considered a special type of natural conduit. Abundant graft material with limited loss of function is the advantage of tendon grafts for nerve bridging [50].

Another natural nerve conduit is represented by acellular nerve grafts, which are endogenous nerve segments in which all the cellular components and the immunogenetic elements have been removed in order to maintain the basal lamina and extracellular matrix which play an important role in repairing peripheral nerves [3,28]. These conduits retain their natural physical, chemical, mechanical, and spatial aligned architecture. Consequently, their use as a graft can promote more physiologic proliferation, differentiation, attachment, migration, and bioactivity, ensuring a homogeneous distribution of MSCs [3]. Acellular conduits are promising candidates to mimic an ECM microenvironment and to support nerve regeneration and cell differentiation; their limited availability is still a challenge for their wide-scale implementation [8].

A wide variety of natural biomaterials such as gelatin, collagen, chitosan, fibrin, etc. can be used for nerve gap bridging [8]. Until now, collagen has been one of the most used natural materials [8], although recent studies highlighted some limitations linked to its use [3]. In particular, collagen-based nerve conduits are the most investigated ones but, their rigid texture, high cost, and inability to bind the surrounding cells make them barely applicable [3].

##### Artificial Nerve Conduits

The development of artificial nerve guides is justified by the limited availability of autologous nerve grafts and donor nerve morbidity. Several types of artificial nerve conduits have been studied to find more performing materials that can replace natural autologous conduits in repairing nerve lesions, especially in select cases with severe nerve injuries and loss of substance. They provide a patent lumen space for sprouting axons and prevent scar formation [25].

Nonbiological conduits can be divided into two categories: absorbable (or biodegradable) and nonabsorbable [27].

Among the nonabsorbable materials, silicon tube and elastomer hydrogel are the most cited. These artificial materials have the disadvantage of engendering chronic foreign body reactions due to scar tissue formation, inflexibility, and lack of stability [50].

Conduits made from different synthetic absorbable polymers offer several advantages: the possibility of attaching Schwann cells or bioactive molecules and delivering them during biodegradation. However, they did not enhance the in situ differentiation potential of the transplanted stem cells: in fact, only a few studies reported the spontaneous trans-differentiation of MSCs into Schwann cells [8].

Biodegradable synthetic polymers are advantageous because of their flexibility, biocompatibility, degradation behavior, porosity, and mechanical strength [50].

The most used biodegradable polymers are poly-3-hydroxybutyrate (PHB), microstructured poly-caprolactone (PCL), poly (dl-lactide-e-caprolactone), polyglycolic acid, silk fibroin, silicone tube, and polytetrafluoroethylene. Either alone or in combination with stem cells, they promote nerve regeneration, myelination, and reinnervation [7,8].

Polyglycolic acid (PGA), one of the most used synthetic biodegradable polymers, reduces the risk of nerve compression and fibrosis associated with nondegradable conduits [3].

Nerve guides should permit the diffusion of neurotrophic and neurotropic factors derived from the distal nerve stump to mimic autologous nerve graft properties [25].

A promising next step in the future of nerve conduits is the use of synthetic polymers and naturally occurring ECM proteins to create an intrinsic framework that can effectively guide regenerating axons [7,24,27]. A new class of polymers that allows electrical conduction—and therefore nervous impulse—is currently being screened as a new frontier for the creation of conduits for stem cells delivery [3].

In any case, the addition of factors such as extracellular molecules, RGD tripeptide (Arg-Gly-Asp), vascular endothelial growth factor (VEGF), and bFGF has given better results, allowing its use [32].

#### 3.2.3. Delivery Modalities

It is important to consider the strategy used to deliver stem cells into the nerve conduit lumen. This can be accomplished in a number of ways: cells can be suspended in medium and injected into a hollow nerve conduit; suspended in a supportive matrix, which is then injected into the lumen of a hollow nerve conduit; or cocultured directly in or on biomaterials used to fill the lumen of a complex nerve conduit [27].

The first technique, where cells are suspended in medium [1,2,4,15,20,22,28,34,51] and seeded directly into a hollow nerve conduit design, is the simplest and well-studied method of cell delivery; it provides the least structural support for transplanted cells [27].

Soaking nerve grafts, in which the nerve samples are pretreated with a micro-needle roller in MSC solutions, is another described method to deliver a higher number of stem cells in the outer zone. This dynamic seeding has been successful in vascular tissue engineering and resulted in a more efficient and uniform distribution of cells compared to static seeding [10].

The second technique, by which cells are suspended in a matrix [3,16,24,26,31] and injected into nerve conduits, provides the advantage of tailoring the extracellular environment to improve cell viability at the cost of simplicity [27].

The third approach for stem cell delivery, where cells are grown and integrated with the nerve conduit prior to transplantation, is the most complex and time consuming but offers the greatest potential for tissue engineering [27].

Another novel strategy for increasing nerve guidance channel (NGC) performance is represented by a cell-sheet system: for instance, MSC sheets consist of an organized row of MSCs immobilized in an aligned collagen conduit capable of rehabilitating nerve guidance and neurite elongation [3].

### 3.3. MSC Differentiation

As mentioned in the Introduction of this paper, mesenchymal stem cells have to be differentiated. Many authors demonstrated that this cellular behavior is mediated by several inflammatory or chemotactic factors [18]. No differences were found in the factors promoting differentiation for different types of mesenchymal cells, as shown in the following paragraphs.

However, there are differences in the growth factors used for the differentiation of mesenchymal stem cells based on the need to differentiate into Schwann-like cells or the neuronal-like phenotype, as explained in Section 3.3.2 and Section 3.3.3.

#### 3.3.1. Paracrine Role of MSCs

MSCs show a strong paracrine potential, and their secretome can be responsible for nerve regeneration [30]. Indeed, MSCs can stimulate proliferation and differentiation of different cell types [18]. Cell-to-cell contacts and paracrine signaling modulate the active molecule secreting capabilities of stem cells and asynergistically induce the secretory activity of endogenous Schwann cells and macrophage accumulation near the site of injury [8].

It was demonstrated that the release of growth factors, cytokines, and interleukins can also influence MSC migration via an autocrine loop for the expression of Aquaporin 1 and CXCR4, of which the levels are increased thanks to activation of the Akt and Erk intracellular signaling pathways [18]. Moreover, the MSC secretome can also exert immunomodulatory, anti-inflammatory, neurotrophic/neuroprotective, and angiogenetic effects on the host microenvironment, thanks to the expression of the major histocompatibility complex-I) tumor necrosis factor (TNF) ß1, interleukin (IL)-13, IL-18 binding protein, ciliary neurotrophic factor (CNTF), and neurotrophin 3 factor (NT3) [18].

Stem cells can assist in peripheral nerve regeneration by preparing an augmented neuroprotective microenvironment that prevents nerve degeneration and apoptosis and supports neurogenesis, axonal growth, re-myelination, and cell metabolism [18] that consist of neurotrophin-3 (NT-3) [18], neurotrophin-1 (NT-1) [18], neurotrophin-4 (NT4) [10], ciliary-derived neurotrophic factor (CDNF) [18], brain-derived neurotrophic factor (BDNF) [3,10,18], nerve growth factor (NGF) [18], GDNF4 [18], bFGF [18], ad CNTF [10].

To increase neurovascularization, MSCs can also secrete the tissue inhibitor of metalloproteinase-18, VEGF [10,18], angiopoietin-1 [3], IGF [3,18], platelet-derived growth factor (PDGF) [18], IL-6 [18], IL-8 [18], transforming growth factor-β (TGF-β) [10], and HGF [3,10,18]. Indeed, the incorporation of VEGF eluted in microspheres and released in MSCs has neurotrophic and mitogenic effects on peripheral nerves, promoting axonal growth and Schwann cell proliferation following trauma [44].

Finally, MSCs can also promote neuronal proliferation and survival by inhibiting the inflammatory responses and proapoptotic pathways: these represent crucial steps for inducing tissue regeneration [33].

#### 3.3.2. Targeted Stimulation of MSCs to Achieve Differentiation in Schwann-Like Cells

Schwann cells play a key role during peripheral nerve regeneration and are responsible for myelin sheath formation around peripheral nerve fibers. After nerve injury, Schwann cells produce several extracellular molecules, such as laminin and type IV collagen that support and provide guidance for axonal elongation [37]. At this purpose, differentiation of autologous MSCs from patients into Schwann-like cells could represent an intriguing approach to improve the regenerative environment [52].

To this aim, many authors tried to develop several in vitro differentiation protocols. However, the real occurrence of glial differentiation of MSCs is still currently much debated, since the majority of these works are simply based on morphological evidence and glial marker expression [53].

Anyway, the most representative protocol of MSC neural induction to a Schwann cell-like phenotype includes a preparation steps with β-mercaptoethanol (β-ME) and all trans retinoic acid (RA) [7,10]; then, the differentiation process is performed using several growth factors including PDGF, bFGF, forskolin (FSK), and Neuregulin-1 β [7,8,10]. This growth factor cocktail seems to be able to induce in 2-week MSC trans-differentiation, since cells display an elongated morphology and are immunopositive for glial-specific markers such as GFAP, S-100, and p75 according to the Schwann-like cells phenotype.

Another protocol to differentiate MSCs in Schwann-like cells could also be achieved by electrical stimulation that alters cellular membrane potential. It has been reported that, after electrical induction, more than 80% of MSCs are immunopositive for S-100 and p75. Furthermore, these cells enhance the production of NGF. Despite the encouraging results achieved by these differentiation techniques, protocols focused on physical methods remain unclear and still need to be tested [7,8,10].

#### 3.3.3. Targeted Differentiation of MSCs to Neuronal-Like Phenotype

Many authors also claim the possibility for MSCs to acquire neuronal antigens, although this could simply reflect their extreme immaturity and their undetermined fate [53]. This was further suggested by the lack of trains of action potentials or synaptic activities in neuron-like MSCs [18].

However, the possibility to induce in vitro MSCs to differentiate into neuronal phenotype still intrigues researchers in this field.

Among the experimental protocols proposed, the administration of bFGF and epithelial growth factor (EGF), which are both mitogenic factors, seems to induce the formations of cellular spheres that are then cultured in the presence of BDNF and all-trans-retinoic acid. After four weeks of culture, cells display immunoreactivity for neuronal markers such as NeuN and Nestin and a presumed neuronal morphology [10].

Yu et al. developed another protocol to induce BMSC differentiation into the neuronal phenotype based on a pre-induction with Forskolin (an adenylate cyclase activator) and fibroblast growth factor-2 (FGF2) for 24 h, followed by the administration of sonic hedgehog and retinoic acid. This approach allowed the appearance of glutamatergic neuron markers, including VGluT1 (type I vesicular glutamate transporter), calretinin (calcium binding protein), and P2 × 3 (an ATP-regulated ion channel receptor) as well as NeuN, MAP-2, and GluR4 [36].

Electrical stimulation to induce neuronal differentiation is another promising approach, but it remains an unexplored field since its practical benefits remain unclear compared to differentiation with growth factors [10]. It has been supposed that differentiation could result from the upregulation of specific signaling pathways including the focal adhesion kinase (FAK) or mitogen-activated protein kinase signaling (p38), MAPK, PI3K, ROCK, and ERK pathway as well as alteration of the cellular membrane potential via hyperpolarization and/or depolarization, modification of ion channels, calcium channel activation, or the increase in intracellular reactive oxygen species (ROS) generation [8].

Finally, electromagnetic fields (EMF) induce NADH oxidase activation that generates ROSs at the level of the plasma membrane. The increased ROS levels further phosphorylate EGFR, which in turn leads to CREB activation through the PI3K/Akt pathway. Uz et al. hypothesized that EMF-induced CREB phosphorylation could promote neuronal differentiation of BM-MSCs [8].

Although MSCs apparently show a neurogenic transformation in vitro, the majority of in vivo studies do not show direct differentiation of the transplanted ADSCs into neurons [39]. Indeed, as previously stated, many authors ascribe the regenerative capacity of MSCs to their paracrine role rather than to a trans-differentiation mechanism. This effect is more likely due to the secretion of neurotrophic factors by ADSCs. Several studies have shown that certain neurotrophic factors (such as BDNF, NGF, and GDNF) are elevated in the conditioned media of MSC cultures. Presumably, MSCs are capable of inducing intrinsic healing employing the host cells under the orchestration of resident Schwann cells. Additionally, the role of paracrine factors in the immunosuppressive effects of MSCs must be considered [30].

## 4. Clinical Considerations

In the previous sections, different MSCs, growth factors, and techniques of stem cell delivery were analyzed. The amount of data and the great heterogeneity of experimental models in the literature reflect the interest of research for the treatment of nerve injuries to optimize medical and surgical options available to date [54]. However, beyond the great potential of these cell-based therapy, the clinical applications of MSCs display some relevant ethical limitations, for instance, the requirement of careful regulatory procedures and the possibility of clinical side effects.

Because of the wide range of possible alternatives, information exchange between medical doctors and researchers in this field is crucial. For example, surgeons are often not aware of specific conduit characteristics when performing nerve repair [15,55], which could lead to missing the best treatment option for a specific case, narrowing the complete view of treatment possibilities in the case of a nerve injury.

To date, autologous nerve grafting is still the method of choice for bridging peripheral nerve gaps [15,19], but promising results have been derived from other options deepened by the studies reviewed in this paper (see Table 3).

For this paper, 15 studies employing different types of mesenchymal stem cells were considered. All except Grimoldi et al. (2015) were experimental animal model studies, mostly involving rats, with iatrogenic sciatic nerve lesions (Eren et al., 2015 [25] used a peroneal nerve lesion). Considering only the rat studies, each study followed the same procedure: different lengths of lesions (0 mm, transected or crushed [4,17,35]; 5 mm [22]; 6 mm [20]; 10 mm [1,15,24,28]; and 15 mm/16 mm [25]) were performed, mesenchymal stem cells were inoculated with different modalities, and different outcomes were monitored through different methodologies which will be discussed later in Table 3. The optimal gap was definitely the smallest one [4,17,35] because it is easier to recover. It can be observed how the gap to be filled and the distance lesion-target correlate with the useful time to the lesion recovery.

The distance between the stumps of the nerve is not the only significant factor conditioning nerve recovery since this mechanism is importantly influenced by injury characteristics: for example, a nerve can be transected (neat cut) or stretched after a traumatic injury. Through a comparison with the studies in Table 3, it can be asserted that the best outcome (in terms of speed of recovery and regeneration) occurs when the nerve injury is neat, without stretching of the nerve. The related studies, however, were carried out on iatrogenic injuries; hence, this bias is cancelled because none of these lesions are the result of accidental trauma and stretching injury is marginal in these cases.

The type of lesion, transected [4,35] or stretched [17], is related, instead, to the quality of the outcome, whatever it may be. Usually in the transected injury, there is a reduced inflammatory response compared to the stretched injury; this favors nerve regeneration in the first case compared to the second. However, in the studies under examination, it was not possible to draw a comparison of the therapeutics success, as they were taken into account using different regeneration parameters and follow-up periods.

The stem cell inoculum was preceded by treatment with growth factors, discussed in depth within Section 3.3.1, Section 3.3.2 and Section 3.3.3, and consisted in different delivery methods (see Table 3).

In nonhuman studies, the maximum follow-up period ranged between 2 [24] and 12 weeks [15].

The optimal follow-up period depends not only on the gap of the lesion but also on the distance between the lesion and the muscle. Considering more than 2.2 mm of growth per week of the nerve [31], an amount of 3 weeks to resolve a lesion of 10 mm could be assumed. This period would stretch in the case of multiple lesions and non-direct suture on the stump, but it would be recovered with nerve reconstruction and graft suture with distal and proximal stump (8 weeks and 12 weeks).

The effects of treatment were then evaluated according to the experimental protocol using different techniques: motor function/reflex measurements, electrophysiological study, immunohistochemistry, MRI, EMG, SFI SSI, muscle weight, and histological analysis.

The electrophysiology tests [4,15,25,34,45] were the first ones to be positive for signs of reinnervation of the explored district, which are useful also in guiding the timing of the follow up. The most used method is EMG. It anticipates any clinical and motor evaluation. MRI [1,35,45] is very useful when used preoperatively to assess the injury, but it has limited use postoperatively. Nevertheless, it may have advantages in the case of lesions on nerves of greater diameter (e.g., brachial plexus, sciatic nerve, etc.). Histological tests [1,4,17,19,20,28,31,34,35] are very useful intraoperatively to orient the surgeon and to assess cell differentiation capabilities at an experimental level.

Different types of outcomes have been analyzed, both clinically and morphologically. Even though some of them are mostly used for recovery evaluation, the time elapsed from treatment is not standardized among the studies, adding further confusion.

Anatomical/histomorphometric measurements seem to be more comparable to each other, but the correlation between morphological and functional data is usually poor [15,56,57,58].

To summarize the results of the reviewed studies, apparently the following conclusions can be stated from the outcomes. Peripheral nervous system lesions treatment with mesenchymal stem MSCs results in improvement in regeneration of the lesion and in functionality of the affected limb, both in terms of movement and sensitivity; however, both classical and newly developed methods of assessing nerve recovery do not always predict a motor and sensory recovery [15]: even a direct comparison between modern studies could not prove significant clinical evidence of the best treatment option. By analyzing the studies in Table 3 it is not easy to immediately identify an MSC group more effective than the others for the treatment of peripheral nerve lesions. In our opinion, more standardized methods of study and outcome evaluation are demanded to get a more direct comparison of the results. Indeed, examining Table 2 and summarizing the main characteristics of the different MSCs, ADSCs emerge as the most suitable MSCs to be used in the case of peripheral nerve injury (see Section 5. Conclusions).

Another bias in nerve injury evaluation (and their recovery) is the vascular damage that often accompanies these processes [2]. Hobson [59] showed that administration of VEGF in a nerve conduit used to treat a 1-cm sciatic nerve gap enhanced axonal regeneration with 78% more myelinated axons after 6 months. Vascular recovery in the treatment of nerve injuries is still unclear, as are the exact mechanisms of MSCs in improving functional outcome in these traumatic cases [2]. If the vascular damage influences nerve regeneration, both these mechanisms appear to be related to nerve recovery and, in this way, directly to clinical recovery [34].

## 5. Conclusions

As suggested in the previous paragraph, there is a lack of comparative studies among the different types of MSCs. The purpose of this review was to identify and propose the MSC type most suitable for clinical use based on preclinical evidence (Table 2). Among the different MSCs analyzed, ADSCs and BMSCs seem to better support and stimulate axonal growth after a PNI in an equivalent manner. Moreover, compared to the other types, both ADSCs and BMSCs had the greatest production of paracrine factors [7,30,32,33,38,40,41,60]. BMSCs were the first cells to be identified and studied in regenerative medicine, but the presumed differentiation potential of ADSCs, their extremely low immunogenicity, their high survival rate, their ease to be obtained with noninvasive procedures, and their availability in large amount lead to their consideration among the most promising for clinical use in PNI (see Table 2).

These considerations should guide future studies in the field of PNI. Potential confirmations will show up in the near future from clinical trials. Indeed, a clinical trial aiming to investigate the safety and efficacy of autologous adipose-derived mesenchymal cell (ADSC) transplantation in individuals with failure to reconstruct peripheral nerves is ongoing. ADSCs will be used during a last-chance surgery (neurolysis and nerve release) on a previously reconstructed nerve. All enrolled patients will undergo documented clinical and electrophysiological observation of the last 2 years. Each patient will receive once 10 microinjections of ADSC along the injured nerve directly after nerve neurolysis. Safety, adverse events, and efficacy will be confirmed by clinical, elecrophysiological (EMG and sensory threshold), and DASH (Disabilities of the Arm, Shoulder and Hand) surveys (ClinicalTrials.gov identifier: NCT04346680).

In this scenario, a tight collaboration between biotechnological research institutes and medical centers could represent a florid background to reach significant results. Before completely understanding which source of stem cells (potentially, ADSCs) and the combination of growth factors and conduit types could represent the best option to aid surgery or medical therapies in functional nerve repair, further studies should be carried out to understand the optimal way to compare clinical outcomes, histomorphometric measurements, and their correlation in animal and in human models. Additional analyses are necessary to pave the way for a more complete understanding of these particular and delicate processes.

## Figures and Tables

**Table 1:** Article subdivision depending on the type of mesenchymal stem cell taken into account. ADSC: adipose stem cells; BMSC: bone marrow stem cells; FetalSC: fetal stem cells; DPSC: dental pulp stem cells. Studies with more than one stem cell source are reported in the final column.

ADSC	BMSC	FetalSC	DPSC	MSC (More Than One Source)
Tremp et al., 2015 [1]	Mohammadi et al., 2014 [15]	Matsuse et al., 2010 [16]	Zhang et al., 2016 [17]	Cofano et al., 2019 [18]
Resch et al., 2019 [19] Santiago et al., 2009 [20]	Chen, et al., 2016 [21]	Pan et al., 2006 [22]	Carnevale et al., 2016 [23]	Yousefi et al., 2019 [3]
Di Summa et al., 2010 [24]	Eren et al., 2015 [25]	Pan et al., 2007 [26]		Flores et al., 2017 [27]
Liu et al., 2011 [28]	Sullivan et al., 2016 [9]	Moattari et al., 2018 [4]		Kemp et al., 2008 [29]
Widgerow et al., 2013 [30]	Dezawa et al., 2001 [31]	De Albornoz et al., 2011 [5]		Jones et al., 2016 [32]
Wang et al., 2019 [33]	Dadon-Nachum et al., 2011 [34]			Yang et al., 2020 [35]
	Yu et al., 2019 [36]			Jiang et al., 2017 [7]
	Matthes et al., 2013 [2]			Mathot et al., 2019 [10]
				Sebben et al., 201 [37]
				Uz et al., 2018 [8]
				Kubiak et al., 2019 [38]

**Table 2:** Mesenchymal stem cells (MSCs) characteristics. Adapted from Cofano et al., 2019 [18].

	Availability [24,28,30,32,37,38,39,40]	Invasive Procedure of Collection [24,28,30,32,37,38,39,40]	Paracrine Growth Factors [7,30,32,33,38,40,41,42]	Immunogenicity [16,28,33,38,41,43]	Use in Pre-Clinical Studies [1,2,4,15,17,19,20,22,23,30,31,35,44]	Axonal Growth [1,15,20,22,31]	Survival [7,20,27,28,32,38]
BMSC	++	+++	+++	/	++	+++	/
ADSC	+++	+ (minimally invasive)	+++	---	+++	+++	++
UMDSC	++	Not invasive	+	Immunologically inert	+	/	/
AMSC	++	Not invasive	+	--	+	++	+
DPSC	+	+	++	--	+	/	+
SKSC	+	+	/	/	/	/	/

The common characteristics studied for each type of mesenchymal stem cell have been reported. This table gives an overview of the specific characteristics and allows a comparison of the various types in order to give a clear and concise indication. ADSC: adipose stem cells; BMSC: bone marrow stem cells; UMDSC: umbilical cord-derived mesenchymal stem cells; AMSC: amniotic fluid mesenchymal stem cells; DPSC: dental pulp stem cells; /, not reported; +, high; ++, very high; +++, extremely high; --, very low; ---, extremely low.

**Table 3:** Main studies regarding the use of MSCs in nerve repair.

Cell Type	Differentiated Cell Type/Differentiation Factor	Animal Nerve Model	Type of Procedure	Nerve Gap	Postoperative Time	Analysis	Results	Reference
BMSC	Astrocyte-like	Rat sciatic nerve	Intramuscular injection		3 weeks	Motor function rotarod test, lateral reflex measurements, electrophysiological study, immunohistochemistry	Increased motor performance, full reflex response, CMAP, and conduction latency were restored in the treated group.	Dadon-Nachum, 2011 [34]
BMSC	Schwann cell-like	Rat sciatic nerve	**Group 1**: artificial grafts (Matrigel) + cells Group 2: artificial graft (Matrigel)		3 weeks	Immunohistochemistry	In the differentiated MSC graft, the distance of regrowth was 2.2 mm at 1 week and reached up to 8 ± 10 mm at 3 weeks, whereas in the undifferentiated MSC graft, only a growth of 2.5 mm at 3 weeks was achieved.	Dezawa, 2001 [31]
BMSCs		Rats Rightperoneal nerve	**Group 1**: nerve excised—saline filled vein graft **Group 2**: nerve excised, reversed and used as an autogenous nerve graft. **Groups 3–6**: nerve discarded	15/16 mm	8 weeks	Gait analysis, PFI, axon counts, EMG	For PFI and EMG, no statistical differences between group 2 and 5 were found. For axon counts, no statistical differences between 2 and 5 and between 5 and 6 were found.	Eren et al., 2015 [25]
BMSC		Rat	Sham-operated group (SHAM), sciatic nerve transection group (SNTG), Artery graft (IOAG)	10 mm	4–8; 8–12 weeks	Sciatic functional index (SFI), Static sciatic index (SSI), Electrophysiological measurement	Nerve conduction velocity in BMSC-treated animals was significantly higher than that in the IOAG group.	Mohammadi, 2014 [15]
BMSC		Rat	injected femoral vein		3 weeks	Determination of the walking track with analysis of the sciatic functional index	The locomotor improvement was observed in 14 days. The functional improvement in the MSC group was significant in 7 days, but the rate of change in improvement from 14 to 21 days.	Matthes, 2013 [2]
ADSC		Rat Sciatic nerve	**Group 1**: ANA injected with ADSC **Group 2**: ANA injected with DMEM medium	10 mm	12 weeks	SFI, electrophysiological study, muscle weight measurement (anterior tibial muscle), histological examination, tissue preparation, immunofluorescence staining	The SFI of the ADSC group was significantly improved compared to the DMEM group, but there was no obvious difference in comparison with the autograft group Histological examination showed regeneration of the nerve tissue in the ADSC group.	Liu, 2011[28]
ADSC	Schwann cells	Rat sciatic nerve	**Group 1**: fibrin conduit + ADSC **Group 2**: fibrin conduit + MSC **Group 3**: fibrin conduit + Schwann cells	10 mm	2 week	Quantification of regeneration length	In the short term, the fibrin conduit can optimize peripheral nerve regeneration. ADSCs promote regeneration in the same manner as MSCs.	Di Summa, 2010 [24]
ADSC	Schwann cells	Rat	**Group 1**: nerve conduit + APCs **Group2**: conduit **Group3**: autograft **Group4**: empty	6 mm	3 weeks	SFI, immunohistochemistry, gastrocnemius muscle weight ratio, histological analysis	The best SFI improvements were observed 3 weeks after surgery in group 1. No difference was observed among groups after 12 weeks.	Santiago, 2009 [20]
ADSC, Schwann cells		-	Co-culture of human Schwann cells and ADSCs on spider silk scaffold	-	3 weeks	Microscope analysis, immunochemistry	Early cell was attached to the spider silk fibers (within 24 h). ADSCs and Schwann cells migrated and proliferated equally along the silk fibers. Spider silk fibers in a long-distance peripheral nerve gap enhance Schwann cell migration.	Resch, 2019 [19]
human-ADSC, rat-ADSC, Schwann cells	FSK, FGF, GGF, PDGF	Rat sciatic nerve	fibrin conduit filled with cells **Group 1**: control **Group 2**: r-ADSCs **Group 3**: h-ADSCs (deep layer) **Group 4**: h-ADSCs (superficial layer) **Group 5**: r-Schwann cells-like cells **Group 6**: h-Stromal Vascular Fraction (SVF) **Group 7**: r-Schwann cells	10 mm	2–4 weeks	MRI, immunocytochemistry	A longer regeneration distance in G7 and inferior results were seen in G4 and G6. A strong correlation between the length of the regenerating axon front measured by MRI and the one measured by immunocytochemistry was observed.	Tremp, 2015 [1]
Wharton jelly stem cells		Rat sciatic nerve	**Group 1**: no intervention **Group 2**: membrane + cells **Group 3**: NGF **Group 4**: NGF + cells **Group 5**: NGF + membrane **Group 6**: NGF + membrane + cells	0 mm transected	8 weeks	SFI, hot water paw immersion test, electrophysiological evaluation, histological analysis	The reaction time in the hot-water paw immersion test significantly decreased in the therapeutic groups. Most increased the amplitude in electrophysiological studies in group 6. The mean number of nerve fibers increased significantly in group 2 and group 6.	Moattari, 2018 [4]
amniotic MSC	FBS, FGF	Rat sciatic nerve	**Group 1**: 4-0 silk filled with fibrin glue and Surgicel **Group 2**: 4-0 silk filled with fibrin glue, Surgicel and MSCs	5 mm	8 weeks	Max diameter axons, nerve continuity, disorientation of fibres, fibrotic tissue invasion, ankle kinematics, SFI	Better results were observed in the MSC group.	Pan, 2006 [22]
SDSC	-	human (1 case report) radial and median nerve	**Right (gap 5 cm)**: sural nerve graft **Left (gap 8–10 cm)**: neuragen filled with SDSCs + interposed sural nerve graft	50/100 mm	3 years	MRI, EMG, clinical	SDSCs were able to differentiate into the GFAP astroglial cell type, glia cells, and Schwann cells. Left biceps and triceps: M2. Better sensor and motor conduction in the right side was observed.	Grimoldi, 2015 [45]
Human gingival MSC (GMSC)	EGF, bFGF, BNF	Rat sciatic nerve	**Group 1**: GMSC seeded on GelFoam. **Group 2**: GMSC—derived neural progenitor cells (iNPCs) seeded on GelFoam **Group 3**: GelFoam alone as the control group	0 mm (crushed)	4 weeks	Histological, immunohistochemical, gastrocnemius muscle weight	GMSCs can be directly induced to multipotent and expandable NPC-like cells. GMSCs and iNPCs could differentiate into both neuronal and Schwann cells. iNPCs possess enhanced therapeutic potential to facilitate regeneration of injured peripheral nerves. GMSCs and iNPCs might delay the demyelination process after injury and might promote remyelination.	Zhang, 2016 [17]
MSC		Rat sciatic nerve	**Group A-D**: MSC + LPS + FK506 **Group B-E**: MSC **Group C-F**: PBS control group	0 mm transected	2–4–8 weeks	Groups A–C: MRI, SFI Groups D–F: histological analysis	Group A: more rapid recovery of fibers at MRI and higher SFIs than other groups were observed. Group D: the best axonal regeneration and faster continuity of nerve fibers in 8 weeks were observed.	Yang, 2020 [35]

The table analyses the eight main points of the studies considered: cell type, differentiated cell type and differentiation factor, animal nerve model, type of surgery, nerve gap, postoperative time, and analysis results. ADSC: adipose stem cells; BMSC: bone marrow stem cells; UMDSC: umbilical cord-derived mesenchymal stem cells; AMSC: amniotic fluid mesenchymal stem cells; DPSC: dental pulp stem cells.

## Data Availability

No new data were created or analyzed in this study. Data sharing is not applicable to this article.

## Abbreviations

PNS	Peripheral Nervous System
MSCs	Mesenchymal Stem Cells
IPSCs	Induced Pluripotent Stem Cells
BMSCs	Bone Marrow Stem Cells
ADSCs	Adipose-Derived Stem Cells
DPSCs	Dental Pulp Stem Cells
FetalSCs	Fetal Stem Cells
SkSCs	Skeletal Muscle Stem Cells
GMSCs	gingiva-derived Stem Cells
CMAP	Compound muscle action potential
PFI	Peroneal function indices
EMG	Electromyography
SHAM	Sham-operated group
SNTG	Sciatic nerve transection group
SFI	Sciatic functional index
SSI	Static sciatic index
ANA	Acellular nerve allografts
DMEM	Dulbecco’s modified Eagle’s medium
APC	Adipose precursor cells
FSK	Forskolin
FGF	Fibroblast growth factor
GGF	Glial growth factor (neuregulin-1b1)
PDGF	Platelet-derived growth factor
SC	Stem cell
NGF	Nerve growth factor
FBS	Fetal bovine serum
GFAP	Anti-glial fibrillary acidic protein
GFP	Green Fluorescent Protein
EGF	Epidermal growth factor
LPS	Innate immune system via lipopolysaccharide
FK506	Tacrolimus
PBS	Phosphate-buffered saline
IOAG	Artery Graft^2^
DASH	Disabilities of the Arm, Shoulder and Hand

## References

[1] Tremp M., Zu Schwabedissen M.M., Kappos E.A., Engels P.E., Fischmann A., Scherberich A., Schaefer D.J., Kalbermatten D.F. (2015). The Regeneration Potential after Human and Autologous Stem Cell Transplantation in a Rat Sciatic Nerve Injury Model can be Monitored by MRI. Cell Transplant..

[2] Matthes S.M., Reimers K., Janssen I., Liebsch C., Kocsis J.D., Vogt P.M., Radtke C. (2013). Intravenous Transplantation of Mesenchymal Stromal Cells to Enhance Peripheral Nerve Regeneration. BioMed Res. Int..

[3] Yousefi F., Arab F.L., Nikkhah K., Amiri H., Mahmoudi M. (2019). Novel approaches using mesenchymal stem cells for curing peripheral nerve injuries. Life Sci..

[4] Moattari M., Kouchesfehani H.M., Kaka G., Sadraie S.H., Naghdi M. (2018). Evaluation of nerve growth factor (NGF) treated mesenchymal stem cells for recovery in neurotmesis model of peripheral nerve injury. J. Cranio-Maxillofac. Surg..

[5] De Albornoz P.M., Delgado P.J., Forriol F., Maffulli N. (2011). Non-surgical therapies for peripheral nerve injury. Br. Med. Bull..

[6] Titolo P., Lavorato A., Isoardo G., Vincitorio F., Garbossa D., Battiston B. (2020). Transfer of the peroneal component of the sciatic nerve in total brachial plexus lesion: An anatomical feasibility study. Injury.

[7] Jiang L., Jones S., Jia X. (2017). Stem Cell Transplantation for Peripheral Nerve Regeneration: Current Options and Opportunities. Int. J. Mol. Sci..

[8] Uz M., Das S.R., Ding S., Sakaguchi D.S., Hondred J.A., Mallapragada S.K. (2018). Advances in Controlling Differentiation of Adult Stem Cells for Peripheral Nerve Regeneration. Adv. Healthc. Mater..

[9] Sullivan R., Dailey T., Duncan K., Abel N., Borlongan C.V. (2016). Peripheral Nerve Injury: Stem Cell Therapy and Peripheral Nerve Transfer. Int. J. Mol. Sci..

[10] Mathot F., Shin A.Y., Van Wijnen A.J. (2019). Targeted stimulation of MSCs in peripheral nerve repair. Gene.

[11] Castro-Manrreza M.E., Montesinos J.J. (2015). Immunoregulation by mesenchymal stem cells: Biological aspects and clinical applications. J. Immunol. Res..

[12] Caplan A.I. (2015). Adult Mesenchymal Stem Cells: When, Where, and How. Stem Cells Int..

[13] Caplan A.I., Hariri R.J. (2015). Body Management: Mesenchymal Stem Cells Control the Internal Regenerator. Stem Cells Transl. Med..

[14] Moher D., Liberati A., Tetzlaff J., Altman D.G., Group T.P. (2009). Linee guida per il reporting di revisioni sistematiche e meta-analisi: Il PRISMA Statement. PLoS Med..

[15] Mohammadi R., Vahabzadeh B., Amini K. (2014). Sciatic nerve regeneration induced by transplantation of in vitro bone marrow stromal cells into an inside-out artery graft in rat. J. Cranio-Maxillofac. Surg..

[16] Matsuse D., Kitada M., Kohama M., Nishikawa K., Makinoshima H., Wakao S., Fujiyoshi Y., Heike T., Nakahata T., Akutsu H. (2010). Human Umbilical Cord-Derived Mesenchymal Stromal Cells Differentiate into Functional Schwann Cells That Sustain Peripheral Nerve Regeneration. J. Neuropathol. Exp. Neurol..

[17] Zhang Q., Nguyen P., Xu Q., Park W., Lee S., Furuhashi A., Le A.D. (2016). Neural Progenitor-Like Cells Induced from Human Gingiva-Derived Mesenchymal Stem Cells Regulate Myelination of Schwann Cells in Rat Sciatic Nerve Regeneration. Stem Cells Transl. Med..

[18] Cofano F., Boido M., Monticelli M., Zenga F., Ducati A., Vercelli A., Garbossa D. (2019). Mesenchymal Stem Cells for Spinal Cord Injury: Current Options, Limitations, and Future of Cell Therapy. Int. J. Mol. Sci..

[19] Resch A., Wolf S., Mann A., Weiss T., Stetco A.-L., Radtke C. (2019). Co-Culturing Human Adipose Derived Stem Cells and Schwann Cells on Spider Silk—A New Approach as Prerequisite for Enhanced Nerve Regeneration. Int. J. Mol. Sci..

[20] Santiago L.Y., Clavijo-Alvarez J., Brayfield C., Rubin J.P., Marra K.G. (2009). Delivery of adipose-derived precursor cells for peripheral nerve repair. Cell Transplant..

[21] Monje P.V., Usach V., Soto P.A., Monje P.V., Setton-Avruj P.C. (2018). EGFP transgene: A useful tool to track transplanted bone marrow mononuclear cell contribution to peripheral remyelination. Transgenic Res..

[22] Pan H.-C., Yang D.-Y., Chiu Y.-T., Lai S.-Z., Wang Y.-C., Chang M.-H., Cheng F.-C. (2006). Enhanced regeneration in injured sciatic nerve by human amniotic mesenchymal stem cell. J. Clin. Neurosci..

[23] Carnevale G., Pisciotta A., Riccio M., Bertoni L., De Biasi S., Gibellini L., Zordani A., Cavallini G.M., La Sala G.B., Bruzzesi G. (2018). Human dental pulp stem cells expressing STRO-1, c-kit and CD34 markers in peripheral nerve regeneration. J. Tissue Eng. Regen. Med..

[24] di Summa P.G., Kingham P.J., Raffoul W., Wiberg M., Terenghi G., Kalbermatten D.F. (2010). Adipose-derived stem cells enhance peripheral nerve regeneration. J. Plast. Reconstr. Aesthet. Surg..

[25] Eren F., Oksuz S., Küçükodaci Z., Kendırlı M.T., Cesur C., Alarçın E., ırem Bektaş E., Karagöz H., Kerımoğlu O., Köse G.T. (2016). Targeted mesenchymal stem cell and vascular endothelial growth factor strategies for repair of nerve defects with nerve tissue implanted autogenous vein graft conduits. Microsurgery.

[26] Pan H.-C., Cheng F.-C., Chen C.-J., Lai S.-Z., Lee C.-W., Yang D.-Y., Chang M.-H., Ho S.-P. (2007). Post-injury regeneration in rat sciatic nerve facilitated by neurotrophic factors secreted by amniotic fluid mesenchymal stem cells. J. Clin. Neurosci..

[27] Armaiz Flores A., Wang H. (2018). The Use and Delivery of Stem Cells in Nerve Regeneration: Preclinical Evidence and Regulatory Considerations. Ann. Plast. Surg..

[28] Tong X., Liu G., Cheng Y., Guo S., Feng Y., Li Q., Jia H., Wang Y. (2011). Transplantation of adipose-derived stem cells for peripheral nerve repair. Int. J. Mol. Med..

[29] Kemp S.W.P., Walsh S.K., Midha R. (2008). Growth factor and stem cell enhanced conduits in peripheral nerve regeneration and repair. Neurol. Res..

[30] Widgerow A.D., Salibian A.A., Lalezari S., Evans G.R. (2013). Neuromodulatory nerve regeneration: Adipose tissue-derived stem cells and neurotrophic mediation in peripheral nerve regeneration. J. Neurosci. Res..

[31] Dezawa M., Takahashi I., Esaki M., Takano M., Sawada H. (2001). Sciatic nerve regeneration in rats induced by transplantation of in vitro differentiated bone-marrow stromal cells. Eur. J. Neurosci..

[32] Jones S., Eisenberg H.M., Jia X. (2016). Advances and Future Applications of Augmented Peripheral Nerve Regeneration. Int. J. Mol. Sci..

[33] Wang Y.-H., Guo Y.-C., Wang D.-R., Liu J.-Y., Pan J. (2019). Adipose Stem Cell-Based Clinical Strategy for Neural Regeneration: A Review of Current Opinion. Stem Cells Int..

[34] Dadon-Nachum M., Sadan O., Srugo I., Melamed E., Offen D. (2011). Differentiated Mesenchymal Stem Cells for Sciatic Nerve Injury. Stem Cell Rev. Rep..

[35] Yang Z., Zheng C., Zhang F., Lin B., Cao M., Tian X., Zhang J., Zhang X., Shen J. (2020). Magnetic resonance imaging of enhanced nerve repair with mesenchymal stem cells combined with microenvironment immunomodulation in neurotmesis. Muscle Nerve.

[36] Yu Z., Xu N., Zhang N., Xiong Y., Wang Z., Liang S., Zhao D., Huang F., Zhang C. (2019). Repair of Peripheral Nerve Sensory Impairments via the Transplantation of Bone Marrow Neural Tissue-Committed Stem Cell-Derived Sensory Neurons. Cell. Mol. Neurobiol..

[37] Sebben A.D., Lichtenfels M., Da Silva J.L.B. (2011). Peripheral nerve regeneration: Cell therapy and neurotrophic factors. Rev. Bras. Ortop. Engl. Ed..

[38] Kubiak C.A., Grochmal J., Kung T.A., Cederna P.S., Midha R., Kemp S.W. (2020). Stem-cell–based therapies to enhance peripheral nerve regeneration. Muscle Nerve.

[39] Zhu M., Heydarkhan-Hagvall S., Hedrick M., Benhaim P., Zuk P. (2013). Manual isolation of adipose-derived stem cells from human lipoaspirates. J. Vis. Exp..

[40] Drela K., Lech W., Figiel-Dabrowska A., Zychowicz M., Mikula M., Sarnowska A., Domanska-Janik K. (2016). Enhanced neuro-therapeutic potential of Wharton’s Jelly–derived mesenchymal stem cells in comparison with bone marrow mesenchymal stem cells culture. Cytotherapy.

[41] Shalaby S.M., El-Shal A.S., Ahmed F.E., Shaban S.F., Wahdan R.A., Kandel W.A., Senger M.S. (2017). Combined Wharton’s jelly derived mesenchymal stem cells and nerve guidance conduit: A potential promising therapy for peripheral nerve injuries. Int. J. Biochem. Cell Biol..

[42] Xiao L., Tsutsui T. (2013). Human dental mesenchymal stem cells and neural regeneration. Hum. Cell.

[43] Luo L., He Y., Wang X., Key B., Lee B.H., Li H., Ye Q. (2018). Potential Roles of Dental Pulp Stem Cells in Neural Regeneration and Repair. Stem Cells Int..

[44] Editor D. (2009). A new, Custom-Made Device for Flap Protection in. Microsurgery.

[45] Grimoldi N., Colleoni F., Tiberio F., Vetrano I.G., Cappellari A., Costa A., Belicchi M., Razini P., Giordano R., Spagnoli D. (2015). Stem Cell Salvage of Injured Peripheral Nerve. Cell Transplant..

[46] Park W.S., Ahn S.Y., Sung S.I., Ahn J.-Y., Chang Y.S. (2017). Strategies to enhance paracrine potency of transplanted mesenchymal stem cells in intractable neonatal disorders. Pediatr. Res..

[47] Kingham P.J., Kalbermatten D.F., Mahay D., Armstrong S.J., Wiberg M., Terenghi G. (2007). Adipose-derived stem cells differentiate into a Schwann cell phenotype and promote neurite outgrowth in vitro. Exp. Neurol..

[48] Carnevale G., Pisciotta A., Bertoni L., Vallarola A., Bertani G., Mecugni D. (2020). Neural crest derived stem cells from dental pulp and tooth-associated stem cells for peripheral nerve regeneration. Neural Regen. Res..

[49] Chen C., Hu J., Huang H., Zhu Y., Qin T. (2016). Design of a Smart Nerve Conduit Based on a Shape-Memory Polymer. Adv. Mater. Technol..

[50] Siemionow M., Bozkurt M., Zor F. (2010). Regeneration and repair of peripheral nerves with different biomaterials: Review. Microsurgery.

[51] Duan X., Cheng L.-N., Zhang F., Liu J., Guo R.-M., Zhong X., Wen X.-H., Shen J. (2012). In vivo MRI monitoring nerve regeneration of acute peripheral nerve traction injury following mesenchymal stem cell transplantation. Eur. J. Radiol..

[52] Brohlin M., Mahay D., Novikov L.N., Terenghi G., Wiberg M., Shawcross S.G., Novikova L.N. (2009). Characterisation of human mesenchymal stem cells following differentiation into Schwann cell-like cells. Neurosci. Res..

[53] Boido M., Garbossa D., Fontanella M., Ducati A., Vercelli A. (2014). Mesenchymal Stem Cell Transplantation Reduces Glial Cyst and Improves Functional Outcome After Spinal Cord Compression. World Neurosurg..

[54] Baldassarre B.M., Lavorato A., Titolo P., Colonna M.R., Vincitorio F., Colzani G., Garbossa D., Battiston B. (2020). Principles of Cortical Plasticity in Peripheral Nerve Surgery. Surg. Technol. Int..

[55] Kehoe S., Zhang X., Boyd D. (2012). FDA approved guidance conduits and wraps for peripheral nerve injury: A review of materials and efficacy. Injury.

[56] Kanaya F., Firrell J.C., Breidenbach W.C. (1996). Sciatic Function Index, Nerve Conduction Tests, Muscle Contraction, and Axon Morphometry as Indicators of Regeneration. Plast. Reconstr. Surg..

[57] Dellon A.L., MacKinnon S.E. (1989). Sciatic nerve regeneration in the rat. Validity of walking track assessment in the presence of chronic contractures. Microsurgery.

[58] Shen N., Zhu J. (1995). Application of sciatic functional index in nerve functional assessment. Microsurgery.

[59] Hobson M.I. (2002). Increased vascularisation enhances axonal regeneration within an acellular nerve conduit. Ann. R. Coll. Surg. Engl..

[60] Li Y., Guo L., Ahn H.S., Kim M.H., Kim S. (2014). Amniotic mesenchymal stem cells display neurovascular tropism and aid in the recovery of injured peripheral nerves. J. Cell. Mol. Med..

